# Correspondence

**Published:** 1885-04

**Authors:** 


### CORRESPONDENCE.

NAVASOTA, Grimes Co., Tex.,
January 15, 1885.

*Dr. Thad S. Up DeGraff, Editor of Bistoury,*

DEAR SIR:—In the BISTOURY for January, 1885, I observe an editorial on "Poisonous Beans," in which surprise is expressed on finding the *Palma Christi beans* violently and fatally poisonous. It is a singular fact that this deleterious property is overlooked and not stated by the publishers of the U. S. Dispensatory. As the castor oil, made from these beans, is so mild in its action and so generally used as a laxative or purge for adults and infants, it is remarkable that the beans are poisonous. They will kill mules and horses, as well as children and men. It is not known in what part of the bean this poison is, whether in the germ or the sarcoderma, the inner covering of the pulp, which, in this seed, is very dark colored, although the pulp is white. My impression is that the poison is of an etherial and evanescent character which is dissipated by the heat and boiling processes used in manufacturing castor oil. Sometimes it seemed to me probable that it is in the sarcoderma, or endopleuva, inside the testa or glossy envelope, and that it is only partially developed in the process of manufacturing oil, and that this poison is what makes some castor oil more griping in its operation, and so difficult to be retained by many stomachs as it is possessed of emetic properties. The strong resemblance which the testa covering of the bean bears to a big, full cow, or dog, tick, is what gave name to the bean; *vicinus* being the Latin word for tick. So few know that they have all their lives been swallowing *tick oil!* It was bad enough before, this makes matters worse, more nauseating.

I will now tell some experience about its killing horses and mules. I was living in Louisiana in 1850-63 and there was an inundation by the Mississippi river in 1850 which destroyed crops, so it was necessary in 1851 to buy Western corn, which was furnished ready sacked from New Orleans to the purchasers. A widow living on Tensaw river, Concordia parish, procured several sacks of corn and her sons fed it sparingly to their plow mules and horses at night, without examining or suspecting any harm The next morning three of the animals were dead. They fed more corn to the others and two of them died. They then looked in the corn and found about three quarts of *Palma Christi beans* to each sack of corn. As they did not know the beans were hurtful, one of them came to see me and learned that they were what killed their animals. By my advice the corn was overhauled and all the beans picked out, and no more harm was done. The widow tried, but in vain, to recover damages from the merchant from whom she had purchased the corn and beans. All that could be elicited was, that the corn was brought from a Western state, where many farmers raise the beans for sale and that through carelessness the beans had been mixed in the barn with the shelled corn. It could not have been done from fraudulent motives as the beans are worth more than corn.

When I came to this place in 1866, I learned from an old gentleman that he had a stallion killed by the beans. He was moving about a hundred miles to a place he had bought—himself and family were in a wagon and the stallion was fastened by a rope and leading behind the wagon and a feed trough was swung behind. The old lady became tired riding one afternoon and got out to walk a little. They passed a dwelling and small farm and the old lady saw several stalks of *palma christi* growing in the fence corners, so she concluded she would take a bunch of the seed and plant them at their new home, as the *palma christi* is esteemed by many "as a good thing to have on the place." She broke off a fine large panicle and placed it carefully in the feed box or trough, under the horses nose. He innocently proceeded to eat of the beans, and in a few hours he died in great pain. The lady had no idea the horse would eat them, nor that if he did, they would hurt him.

It is worthy of notice that some years ago I furnished the BISTOURY a paper detailing facts about a barrel of castor oil which turned out to be wine, but when the wine was all drank, a monster fœtus was found embalmed in the barrel, which is now in the museum of the New Orleans Medical College.

Respectfully,

A. R. KILPATRICK, M. D.

OVID, N. Y., Jan. 16, 1885.

*Editor Bistoury:*

I am somewhat of the opinion that the article in the BISTOURY for January under the caption, "Where is the Fault?" is written from a theoretical rather than from a practical standpoint. Dr. Beecher, I doubt not, is a very wise and a very good man; but still I don't admire the advice he gave the boy who applied to him for something to do—although possibly he gave the advice which would have been most readily accepted.

I have had considerable experience with boys during the past thirty years, and am very much inclined to the opinion that the race is degenerating. In fact, we haven't *boys* any more. As soon as the youth puts off his infantile clothes he becomes a young man and puts on *manly* airs, according to the way he understands it. Talk to him about "learning a trade" after he finishes his school, and he would turn away in disgust. He is either going to be a professional man, a counter-jumper or a dude; most of them fill the bill in the latter capacity. At 16 or 18, if he can obtain money, or credit, he is the regular patron of the livery, the billiard saloon, drinking resorts, horse races, etc. This is why he can't *afford* to be an apprentice; and if he is a clerk, he is hardly a *trusty* one.

I remember the time (I am now aged 47) when there *were* boys, and they weren't too good to soil their hands a little in honest toil. They were willing to begin at the bottom of the ladder and work up,—to start on small wages, and *live* on their pay, too—and earn the increase which time and experience gave them.

Now, the heads of mechanical branches can not afford to take apprentices. It is cheaper to hire journeymen than to pay the wages which new beginners demand. This is the way I find it in my business. I have had many apprentices, and I have my *last* one now. He and I shall part in about six weeks. It is a luxury I cannot afford. Big wages and little to do, and that little of an extremely light and delicate nature, is what the average boy likes. Altogether he is a pretty ornament in society, but doesn't tower up very extensively in the matter of real value.

Of course, I am speaking of the average, not of the whole.

I have written this hastily and with no other object than the hope that it may lead you into another train of thought in regard to the boy question.

I may be a little too severe; but I think the boy, for his own good, should be "handled without gloves." Truly yours, O. C. C.

WATERTOWN, N. Y., March 15, 1885.

*Editor Bistoury:*

The comma bacillus of Asiatic cholera as seen in a genuine slide stained with aniline lately received from Calcutta, has an appearance peculiar in itself and no doubt an identical organism different from any other bacilli known or described.

It is exceedingly small, requiring a good $\frac{1}{10}$ obj., at least, to see its exact form in its secondary or matured condition, and requires a very close manipulation to trace the comma tip to its extremity. It is still better seen with a $\frac{1}{16}$ or $\frac{1}{20}$ obj., being only about the size of a micrococcus or 1 u. x $\frac{1}{4}$ u. in size.

Its growth and development can be traced in the slide, as at first, when in the mucous corpuscle, it is regular in shape, long-elliptical or rice-seed-shaped, placed end to end in chains or beads, and, multiplying by transverse division through the middle of each cell till it completely fills the corpuscle, bursting and destroying it, and being scattered in a free state in the fluid it soon takes on its secondary or comma form, having a blunt and enlarged end which seemingly possesses a spore, and a bodo-like tapering extremity opposite, suitable for giving it a peculiar wiggling motion as one might judge.

Its first condition, though more oval or rounded at the ends than most bacilli as well as its mode of growth, is natural enough, and, in its matured condition it still appears to be a regularly formed bacillus.

Whether this is the primary or secondary cause attending cholera, is another question, although as with all other pathogenic bacilli or bacteria the disease in which each grows most profusely, the poison may be absorbed and carried by them, and in effect all the same, but as to the organism existing, it cannot be doubted. J. H. ADAMS.

ELECTRICAL FIREFLIES.—"The endless diversity of uses to which electricity may be put," says a recent English weekly, "received another illustration on Tuesday night at the Court opera at Vienna, where, by the simple expedient of suspending tiny incandescent lamps by fine swinging wires, the effect was produced of swarms of fireflies flitting about a tropical forest. By switches the current is turned off and on at the pleasure of the operator, and the effect, as the artificial fireflies flash and dance in mid air, is said to have been electrical in other than a literal sense."

### Diseases Produced by Drinking Water Flowing from a Graveyard.

The following cases present some points worthy of serious consideration :

H., age 13, female, of previous good health, while a work in a field on October 1st, 1883, just before taking her dinner drank water from a branch which had its origin from the side of a hill that had been used as a burying-ground.

The place at which she drank the water was about one hundred and fifty feet from the source of the branch. About an hour after she had taken the water she complained of being sick, suffered pain in head and very soon began to vomit, and continued until midnight, when a diarrhœa set up, from which she suffered greatly, complaining of aching all over, which continued for three days, when I was sent for to see her.

I found her with pulse 112, temperature 103° F., suffering intense pain in left hip and leg, tenderness on preasure over sciatic nerve on side ; some swelling of the hip.

I gave patient hypdermic injection of morphine, which gave some relief. The morphine had to be continued at intervals to produce rest.

On the third day after I saw her, her mother called my attention to a swelling of the labum majus of the left side. This very soon resulted in a large abscess which discharged profusely. A few days after this she complained of pain in the knee, wrist and cavicular articulations. These showed very little sign of inflammatron at first, but in a few days suppurated. The parotid glands also suppurated. Patient died on the eighteenth day. The temperature and pulse varied very little from what they were when I first saw her. She was given quinine and iodine, and was kept on as nutritious a diet as could be had, consisting of beef, eggs, etc.

There was a sister who drank water at the same time and place who was sick in the same manner, who had a similar abscess on the scalp to the ones related. This patient recovered.

A year previous to this time an older sister, after drinking at this same place, was taken sick in a few hours and had a long and severe spell of typhoid fever ; she had no abscesses as did the others.

The owner of the land through which this branch flows, informs me that whenever he has permitted his stock to pasture in this field they loose their appetite, fail to eat and very soon get poor. He, knowing the bad effect of this water on his stock, had cautioned his children against drinking it.

Did the drinking of this water produce these diseases ? If so, why soould two of the cases, seemingly have pyemia and the other typhoid fever ?—*Atlanta M. and S. Journal.*

